# Breast Milk Macronutrients and Child Growth and Neurodevelopment in a Lactation Cohort in Rural Bangladesh

**DOI:** 10.1016/j.cdnut.2026.109531

**Published:** 2026-09-16

**Authors:** Krysten North, Sophie Driker, Rasheda Khanam, Salahuddin Ahmed, Chloe Andrews, Mandy B Belfort, Sara Cherkerzian, Nabidul H Chowdhury, Tarik Hasan, Sayedur Rahman, Unmesha Roy Paladhi, Amy Lopez Esteban, Carmen Monthe-Dreze, Fahmida Tofail, Abdullah Baqui, Sarbattama Sen, Anne CC Lee

**Affiliations:** 1 Division of Newborn Medicine, Department of Pediatrics, Brigham and Women’s Hospital, Boston, MA, United States; 2 Department of Pediatrics, Harvard Medical School, Boston, MA, United States; 3 Department of International Health, Johns Hopkins Bloomberg School of Public Health, Baltimore, MD, United States; 4 Projahnmo Research Foundation, Dhaka, Bangladesh; 5 Department of Pediatrics, Women and Infants Hospital of Rhode Island, Providence, RI, United States; 6 Global Alliance for Infant and Maternal Health Research, Department of Pediatrics, Warren Alpert Medical School of Brown University, Providence, RI, United States; 7 Department of Pediatrics, Brown University Warren Alpert Medical School, Providence, RI, United States; 8 Nutrition Research Division, International Center for Diarrheal Disease Research, Dhaka, Bangladesh

**Keywords:** breast milk, macronutrient, lactation, Bangladesh, global health

## Abstract

**Background:**

Limited data exist on the relationship between breast milk macronutrient/energy concentrations and child outcomes in settings with high maternal undernutrition.

**Objectives:**

This study aims to determine associations of breast milk macronutrient (protein, fat, carbohydrate) and energy concentrations with child growth and neurodevelopment.

**Methods:**

In this observational lactation cohort of 99 mother–infant dyads from Sylhet, Bangladesh, mothers provided hand-expressed breast milk samples at 2 and 5 mo postpartum. Infant anthropometrics were obtained at birth, 3, 6, 12, and 24 mo; neurodevelopment was assessed at 24 mo using the Bayley Scales of Infant and Toddler Development-III. Macronutrient concentrations were measured using midinfrared spectroscopy. Associations with growth and neurodevelopment *z*-scores were estimated using mixed models and linear regression, adjusted for a priori confounders. Primary outcomes were length-for-age *z*-score (12 mo) and cognitive *z*-scores (24 mo) in relation to 2-mo protein concentration.

**Results:**

Breast milk samples were provided at 2 (*n* = 98) and 5 mo (*n* = 87). We found no significant associations between 2-mo protein and 12-mo length-for-age *z*-scores or 24-mo cognitive *z*-scores. Higher 2-mo fat (g/dL) was associated with lower weight-for-age *z*-scores at 3 mo [*b*: –0.12 (95% confidence interval (CI): –0.24, –0.005)] and lower head circumference-for-age *z*-scores at 3 [–0.20 (–0.32, –0.09)], 6 [–0.19 (–0.31, –0.08)], and 12 mo [–0.17 (–0.29,–0.06)]. Similarly, 2-mo energy (kcal/dL) was inversely associated with 3-mo weight-for-age *z*-scores [–0.01 (–0.03, –0.001)] and head circumference-for-age *z*-scores at 3 [–0.02 (–0.04, –0.01)], 6 [–0.02 (–0.03, –0.01)], and 12 mo [–0.02 (–0.03, –0.01)]. Higher 2-mo carbohydrate (g/dL) was associated with higher motor *z*-scores [1.08 (0.06, 2.09)], and higher 5-mo carbohydrate with higher cognitive *z*-scores [1.10 (0.15, 2.05)].

**Conclusions:**

Protein concentrations were not associated with linear or cognitive outcomes in this cohort. Our findings suggest that future studies targeting infant outcomes may optimally be designed to account for factors beyond macronutrient concentration, such as daily milk volume intake, that may impact infant growth and development.

## Introduction

The WHO recommends exclusive breast milk feeding ≤6 mo of age and continued breast milk feeding with appropriate complementary feeding up to or beyond 2 y [1]. Globally, reported rates of exclusive breastfeeding (EBF) are high in several low- and middle-income countries (LMICs) in Africa and Asia [2]. Exclusively breastfed infants depend almost completely on their mother’s milk to meet their nutritional requirements for optimal growth and brain development.

Breast milk is a complex substrate with many components, including the macronutrients (protein, fat, and carbohydrate) that are needed to support infant metabolic demands and facilitate tissue growth. Concentrations of breast milk macronutrients and energy vary between individuals and throughout lactation, which may affect infant outcomes [3]. Most studies of the relationship between breast milk composition and infant outcomes have been conducted in high-income settings. A recent systematic review identified 57 studies of breast milk macronutrient components and infant growth [4]. Of these, only 15 were conducted within LMICs, and only 3 of these LMIC-based studies measured more than a single milk macronutrient (protein, fat, or carbohydrate) [[5], [6], [7]]. The dearth of data regarding the association between breast milk composition and subsequent growth is a critical gap in our understanding of the relationship between early breast milk nutrition and growth for infants born in LMICs.

Early infancy is a critical window for brain development, establishing a trajectory that influences later health outcomes into adulthood [8]. Both exclusive and prolonged breastfeeding in infancy have been associated with improved neurodevelopment in childhood, but the mechanism of this association has not been fully understood [9]. Higher intake of breast milk as compared with formula during the first months of life has been associated with improved patterns of brain development and maturation [10,11]. However, most research on breast milk composition and neurodevelopment has focused on preterm infants in high-income settings. Almost no previous studies of full-term infants from LMICs have rigorously evaluated the association between specific breast milk components and neurodevelopment. Improved understanding of the relationship between breast milk composition and neurodevelopment may provide an opportunity to enhance brain growth and functions during infancy, a key time for establishing a positive neurodevelopmental trajectory.

The objectives of our study were to determine the associations between breast milk macronutrient and energy concentrations at 2 and 5 mo with *1*) growth at 3, 6, 12, and 24 mo and *2*) neurodevelopment at 24 mo, among a cohort of mothers and infants in rural Bangladesh. We hypothesized that higher breast milk protein concentrations at 2 mo would be associated with higher length-for-age *z*-scores at 12 mo and with higher cognitive *z*-scores at 24 mo.

## Methods

### Study design and population

We conducted a secondary analysis of a longitudinal cohort study of 99 mother–infant dyads at the Projahnmo research site in Bangladesh [12]. The Projahnmo study site in the northeastern area of the Sylhet district of Bangladesh is rural and agrarian, with a per-person gross domestic product of under $2 daily. Breastfeeding is common—88% of mothers continuing to breastfeed their children until 12–23 mo of age [13]. Thirty-four percent of children under age 5 y are stunted [13].

The lactation cohort was nested within the larger Projahnmo pregnancy-birth cohort that enrolled pregnant women before 20 wk of gestation, dated by enrollment ultrasound prior [12]. Women were consecutively enrolled into the lactation cohort from 3 January, 2018 to 4 April, 2018 at a postpartum visit conducted between 42 and 60 d after delivery. They were followed longitudinally with their infants to 24 mo. The procedures for this study were approved by the institutional review boards at Brigham and Women’s Hospital (#2017P000876/PHS) and the National Research Ethics Committee of Bangladesh Medical Research Council (BMRC/NREC/2016-2019/340). Written informed consent was obtained before enrollment in the lactation cohort. The lactation cohort included participants enrolled in the pregnancy-birth cohort who delivered a liveborn infant and planned to exclusively or predominantly breastfeed. We excluded participants with painful breast conditions, such as mastitis or cracked nipples, or who were breastfeeding multiples.

### Milk collection

We collected breast milk at the enrollment postpartum visit (referred to as the 2-mo visit) and a follow-up visit at ∼5 mo. The milk was collected as a clean, hand-expressed, mid-feed sample. Collection time was standardized to midday. Mothers were asked to feed their infant 2–3 h before the study visit to standardize the time since the last feed. At the visit, participants were asked to breastfeed their infants for 3–5 min, detach the infant, wipe the nipple with a cloth, and hand express 15 mL of milk from the breast into a sterile container. The research team froze the human milk samples in 7.5-mL aliquots. Samples were transported from the field to a central laboratory facility in Sylhet using liquid nitrogen, then frozen in –80°C freezers until they were shipped to Brigham and Women’s Hospital in Boston, using dry ice and temperature monitors to maintain an appropriate cold chain. Once there, samples were maintained at –80°C until analysis.

### Milk composition analysis

We used midinfrared spectroscopy to measure breast milk macronutrient content (Miris human milk analyzer) [14]. This method has been validated in previously published milk composition studies and was approved by the United States Food and Drug Administration in 2018 [15]. We used calibration and quality control procedures with weekly quality control checks per manufacturer recommendations to ensure that measured values remained within an acceptable accuracy range [14]. A subset of samples was measured in duplicate. The Miris reports crude protein, fat, and carbohydrate values. Crude protein is determined by estimating the total nitrogen content of a sample, which is then converted by a factor of 6.38. True protein is then determined as 0.8 × crude protein [14,15]. We used true protein concentrations for our analyses. We determined metabolizable energy concentrations as a calculated value using the Atwater general equation [true protein (g/100 mL) × 4 + carbohydrate (g/100 mL) × 4 + fat (g/100 mL) × 9 = energy] [16]. Metabolizable energy is more clinically relevant than the gross energy values reported by the Miris analyzer [17].

### Growth measurements

Infant anthropometrics, including weight, length, and head circumference, were obtained using Intergrowth-21st standardized procedures at birth, 3, 6, 12, and 24 mo [18]. We measured infant weight using digital scales (TANITA BD585 Pediatric Scale; precision: 10 g), length using a length board (Seca 417 portable infantometer and Seca 213 mobile stadiometer; precision: 1 mm), and head circumference using insertion tape (JiVitA measuring tape; precision: 1 mm). All anthropometrics were measured in duplicate, with a third measurement taken if the difference was greater than the minimum acceptable difference defined by WHO [19].

Several infants had their initial anthropometrics measured after 3 d but within the first 2 wk of birth, because of a delay in the birth study visit. We recalibrated the initial weight measurements to approximate a birth weight using an imputation model from a similar South Asian population [20]. We recalibrated initial length and head circumference measures obtained between day 4 and 14 of life based on a linear rate of growth derived from Intergrowth anthropometric tables and day of life at which the measurements were obtained [18].

Infant birth weight-for-age, length-for-age, and head circumference-for-age *z*-scores were calculated for gestational age (GA) and sex using the Intergrowth-21st growth standards [18,21]. Z-scores for anthropometrics obtained at the 3–24 mo visits were calculated using the WHO sex-specific growth curves and adjusted for GA among preterm infants [22]. Our primary growth outcome was change in length-for-age *z*-score at 12 mo (*b* regression coefficient). Secondary outcomes included length-for-age *z*-score at 3, 6, and 24 mo, head circumference-for-age *z*-score at 3, 6, 12, and 24 mo, and weight-for-age *z*-score at 3, 6, 12, and 24 mo.

### Neurodevelopment assessment

We assessed child neurodevelopment at 24 mo using the Bayley Scales of Infant and Toddler Development, 3rd edition (Bayley-III) [23]. The Bayley-III measures language, motor, and cognitive domains. We used a version of the Bayley-III that has been adapted and validated in rural Bangladesh [[24], [25], [26], [27]]. Cultural adaptation of the Bayley-III primarily consisted of picture modification to be appropriate to the local setting while maintaining the original question intent. The Bayley-III was administered by study staff with a master’s degree who had received a 3-wk, rigorous training by a senior psychologist. Data collection was initiated only after attaining ≥95% agreement between testers and trainer (*r* = 0.95, *P* < 0.001) for language, motor, and cognitive domains [28]. On the basis of our team’s prior experience and studies in Bangladesh, we chose to standardize raw Bayley-III scales by generating *z*-scores within the larger Projahnmo cohort for these analyses instead of using age-standardized conversion tables based on Western population norms [29,30]. Our primary neurodevelopment outcome was the Bayley-III *z*-score of the cognitive domain. Secondary outcomes included language and motor *z*-scores.

### Sample size/minimal detectable effect size

Because this study was a secondary data analysis, the sample size was determined by available data rather than an a priori power calculation. To aid interpretation, we estimated the minimum detectable association for breast milk protein concentration and our primary outcomes (length-for-age *z*-score and Bayley-III cognitive *z*-score). With *n =* 99, 80% power, an observed SD of 0.18 g/dL for 2-mo protein, and a Bonferroni-corrected 2-sided *α* of 0.025, the study had adequate sample size to detect a moderate unadjusted association of ∼1.7 *z*-scores (length or cognition) per 1 g/dL higher protein concentration, equivalent to ∼0.31 *z*-scores per 1 SD higher protein concentration.

### Statistical methods

We summarized descriptive statistics for continuous variables as either means and SDs for normally distributed data or medians and IQRs for nonnormally distributed data. Categorical variables were reported as frequencies and percentages. Macronutrient exposures were assessed as continuous concentrations (g/dL or kcal/dL) at each timepoint. Differences in continuous concentrations at 2 and 5 mo were tested using the Wilcoxon rank sum test.

We evaluated linear, quadratic, and cubic specifications for each exposure–outcome association using Bayesian Information Criterion (BIC). As no meaningful nonlinearity was detected (ΔBIC > −2 for all comparisons), breast milk macronutrient concentrations were modeled as continuous linear exposures. We report calculated associations as adjusted or unadjusted unstandardized regression coefficients (*b*) and 95% confidence intervals (CIs) representing the change in mean outcome *z*-score per 1-unit increase in macronutrient concentration (g/dL or kcal/dL). To account for multiple comparisons, we used a Bonferroni-corrected significance threshold of *α* = 0.025 for the outcomes in the 2 primary hypotheses, that higher protein concentrations at 2 mo would be associated with *1*) greater length-for-age *z*-score at 12 mo and *2*) higher cognitive composite *z*-score at 24 mo. All other nutrient-outcome associations and age-specific estimates were considered secondary and are interpreted descriptively. This analysis was exploratory, intended to generate hypotheses for future studies.

#### Growth regression methods

We conducted univariable and multivariable linear mixed regression analyses to quantify the change in infant weight-for-age *z*-score, length-for-age *z*-score, and head circumference-for-age *z*-scores. Mixed models accounted for repeated measures within the same subject and included fixed effects for exposures (continuous macronutrient or energy concentration), time of growth measurement (age in months), and the interaction between the exposure and time. On the basis of our conceptual model in Figure 1, multivariable models were adjusted for potential confounders determined a priori, including wealth index, maternal age, maternal BMI, and *z*-score at birth (weight, length, or head circumference, per the outcome measured). To account for individual differences, we specified a random intercept for each infant with an identity covariance structure. To model the correlation between repeated measurements within the same infant, we specified an exchangeable residual covariance structure, which assumes equal variances at both time points and constant correlation between measurements within the same child. The models were fitted using restricted maximum likelihood estimation with Satterthwaite degrees of freedom.

**FIGURE 1:**
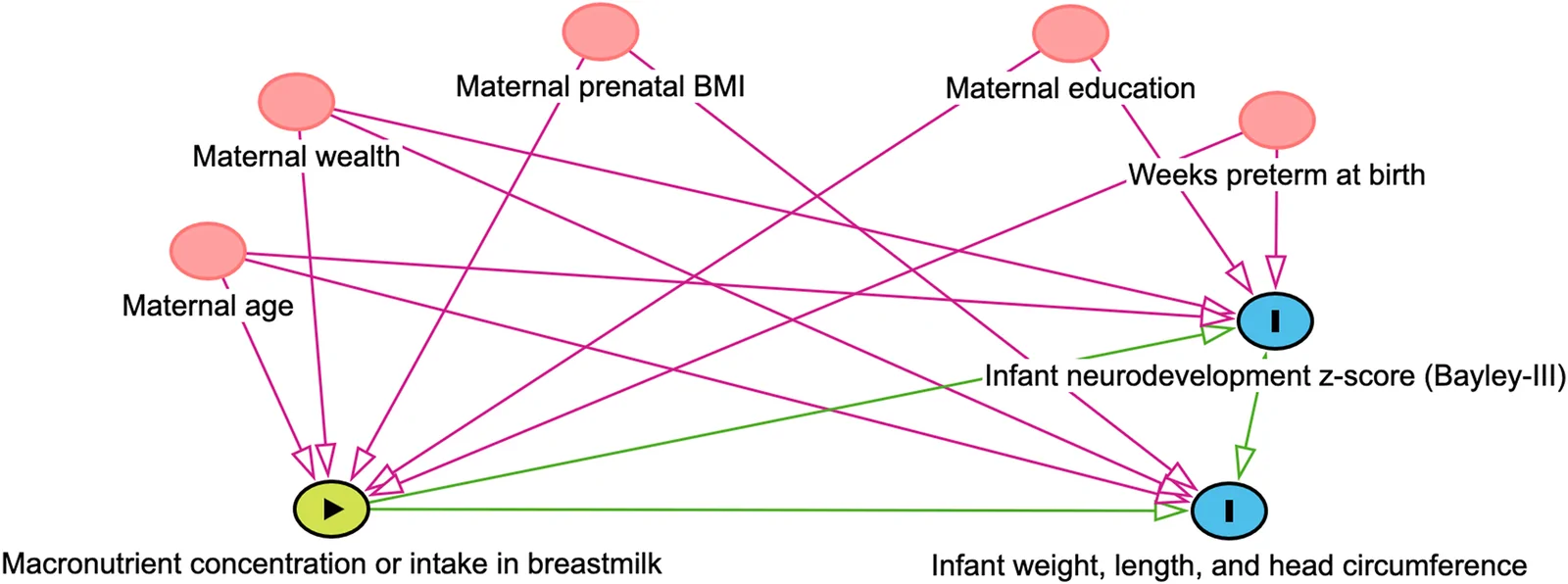
Directed acyclic graph of conceptual model of the associations between breast milk macronutrients and infant outcomes. Green circle = exposures; blue circle = outcomes; pink circles = confounders.

#### Neurodevelopment regression methods

For neurodevelopment, we used univariable and multivariable linear regression to determine the association between macronutrient and energy concentrations (exposure) and Bayley-III *z*-score for language, cognitive, and motor domains (outcome) at 24 mo. On the basis of our conceptual model (Figure 1), multivariable models were adjusted for potential confounders determined a priori: wealth index, maternal education, maternal age, and prematurity.

#### Subgroup analyses

We conducted a subgroup analysis to assess the impact of complementary feeding, limited to infants who were not regularly consuming infant formula, animal milk, or complementary feeds at 5 mo. To test effect modification by EBF, we ran interaction models including both EBF and non-EBF infants for exposure–outcome pairs in which the EBF subgroup analysis suggested statistically different associations in the adjusted models. Interaction testing in the full sample included a 2-way exposure (macronutrient × EBF status) interaction term. Analyses were performed using R Studio version 4.5.2 (Posit Software) [31] and Stata software, v18 (StataCorp LLC) [32].

## Results

We enrolled 99 mothers at 2 mo postpartum who provided breast milk samples at enrollment. Of these, 87 (87.9%) provided a second breast milk sample at the 5-mo study visit. Participant follow-up over time is shown in the flow diagram in Figure 2. Missingness was typically due to loss to follow-up or missed study visits. There were no statistically significant differences in demographics between participants who provided breast milk samples at 2 mo only (*n =* 99) and those who provided samples at both 2 and 5 mo (*n =* 87), shown in Supplemental Table 1. Demographics of lactation cohort participants are shown in Table 1. The sociodemographic characteristics of the lactation cohort were consistent with the parent pregnancy-birth cohort (Supplemental Table 2). Overall, nearly one-third of enrolled mothers were underweight at the start of their pregnancy. The median duration of EBF was 5 mo. At the 2-mo visit, 1 infant was fed with formula, and 2 infants had been fed rice or rice porridge in addition to breast milk. At the 5-mo visit, a small number of infants had received formula (3 infants) or other animal milk (2 infants). Thirty-one infants had been routinely fed solid foods, most commonly rice or rice porridge. As previously reported in this cohort, the protein, fat, and energy concentration of breast milk dropped significantly from 2 to 5 mo (*P* < 0.001; Table 2) [33]. Overall anthropometrics at birth were notable for a head-sparing phenotype, with mean weight *z*-scores of –1.25 but mean head circumference *z*-scores of –0.46. Mean head circumference and length *z*-scores decreased with time, trending to <–1 *z*-scores by 12 mo of age (Figure 3).

**FIGURE 2:**
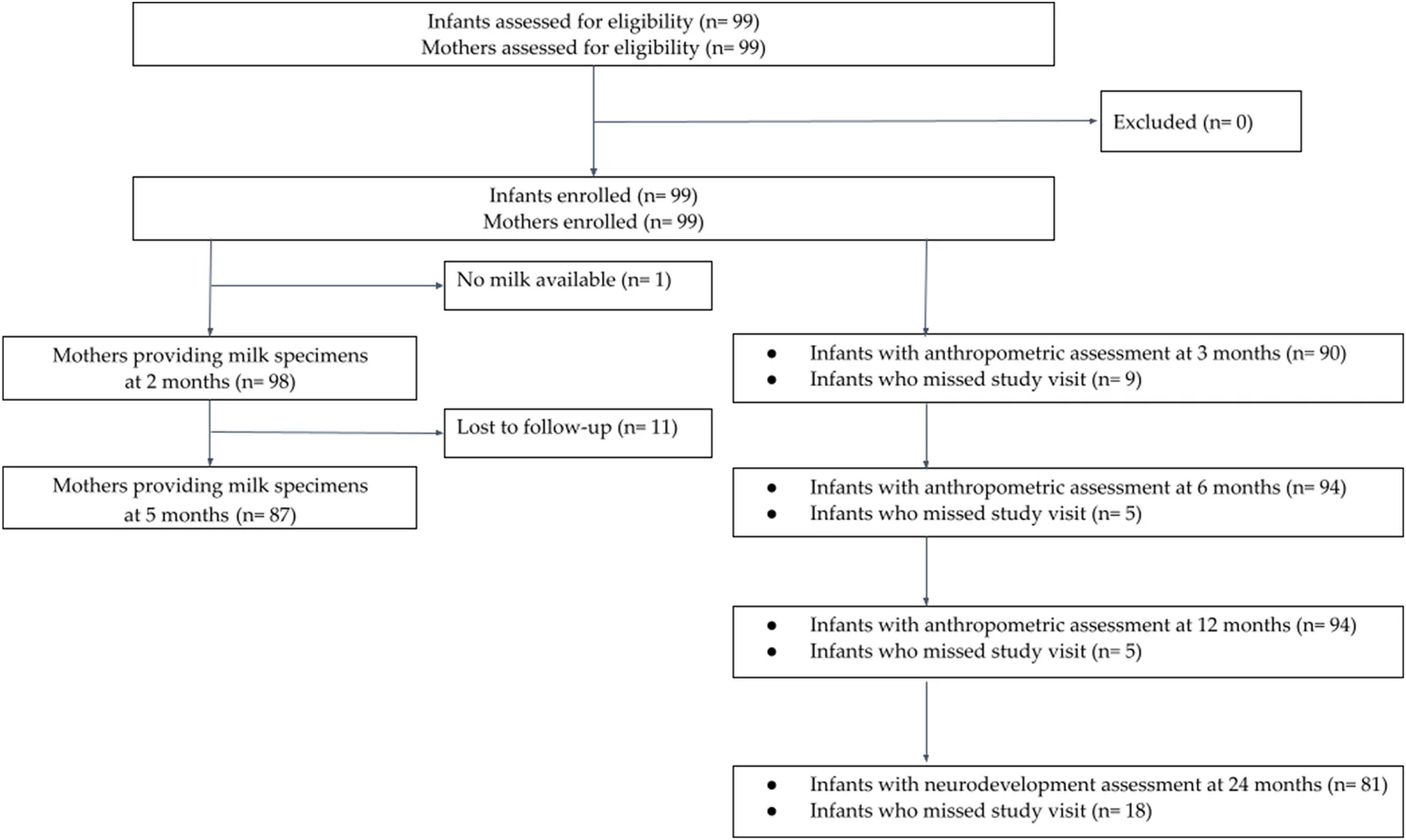
Study participant flow diagram.

**TABLE 1:** Population characteristics of 99 mother–infant dyads

Maternal characteristics	
Age (y)	24.0 [20.0–27.0]
Parity	2.0 [1.0–3.0]
Education (# yr)	6.9 (2.9)
Maternal BMI at enrollment (kg/m^2^)	19.4 [18.0–21.6]
Maternal underweight at enrollment (BMI < 18.5)	31 (31.3%)
Low MUAC < 23 cm	55 (55.6%)
Low maternal height <145 cm	13 (13.1%)
Tobacco or betel nut use in pregnancy	29 (29.3%)
**Infant characteristics**	

Male sex (*n*, %)	50.0 (50.5%)
GA at delivery (wk)	39.6 [38.9–40.4]
Preterm delivery (<37 wk) (*n*, %)	6.0 (6.1%)
Birthweight (g) 1	2747 (358)
Age at introduction of complementary feeds (mo)	5 [4–6]
Infant feeding formula, animal milk, or routinely eating semisolid foods at 2-mo visit	1.0 (1%)
Infant feeding formula, animal milk, or routinely eating semisolid foods at 5-mo visit	31.0 (31.3%)

Continuous values presented as mean (SD) or median [IQR]. Categorical values presented as *n* (%).

Abbreviations: GA, gestational age; MUAC, midupper arm circumference.

1 *n* = 98.

**TABLE 2:** Concentrations of macronutrients and energy in breast milk at each collection timepoint [mean (SD)]

	2 mo	5 mo	Wilcoxon rank-sum (*P* value)
True protein concentration (g/dL)	0.95 (0.18)	0.78 (0.20)	<0.001
Fat concentration (g/dL)	4.68 (1.42)	3.09 (1.35)	<0.001
Carbohydrate concentration (g/dL)	7.85 (0.19)	7.80 (0.23)	0.24
Energy concentration (kcal/dL)	77.31 (12.79)	61.96 (12.27)	<0.001

**FIGURE 3:**
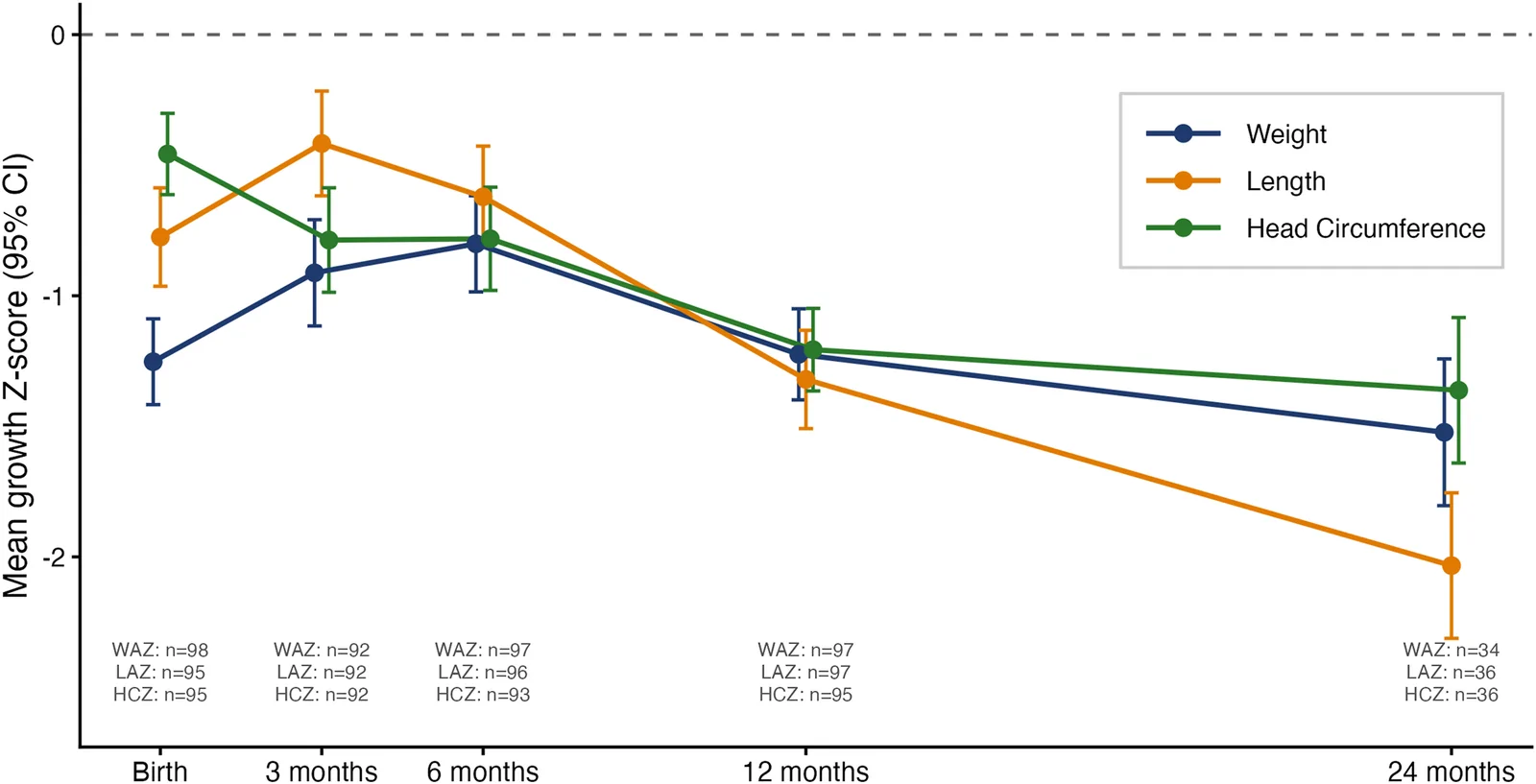
Anthropometric *z*-score mean and 95% CI values at birth, 3, 6, 12, and 24 mo among infants enrolled in this cohort. CI, confidence interval. HCZ, head circumference-for-age z-score; LAZ, length-for-age z-score; WAZ, weight-for-age z-score.

### Macronutrient concentration and growth

We found no association between protein concentrations and length *z*-scores at 12 mo (primary outcome) or any other time point (Table 3). In unadjusted analysis, every additional gram per deciliter of breast milk protein at 2 mo was associated with 1.02 higher head circumference *z*-scores at 12 mo (95% CI: 0.07, 1.98) and 1.19 higher head circumference *z*-scores at 24 mo (95% CI: 0.06, 2.33) (Supplemental Table 3). However, this was not significant in the adjusted model (Table 3).

**TABLE 3:** Adjusted associations between breast milk macronutrient concentration and infant growth *z*-scores

	Weight-for-age *z*-score	Length-for-age *z*-score	Head circumference-for-age *z*-score
	*b* (95% CI)	*b* (95% CI)	*b* (95% CI)
Protein			
2-mo protein (g/dL)	*n =* 96	*n =* 93	*n =* 93
3 mo	–0.05 (–1.01, 0.91)	0.39 (–0.48, 1.26)	0.76 (–0.14, 1.65)
6 mo	–0.07 (–0.99, 0.85)	0.31 (–0.51, 1.13)	0.78 (–0.08, 1.64)
12 mo	–0.11 (–1.03, 0.81)	0.15 (–0.79, 1.08)^1^	0.83 (–0.03, 1.69)
24 mo	–0.20 (–1.36, 0.97)	–0.18 (–1.31, 0.95)	0.92 (–0.14, 1.99)
5-mo protein (g/dL)	*n =* 87	*n =* 84	*n =* 84
3 mo	—	—	—
6 mo	–0.23 (–1.15, 0.69)	0.24 (–0.72, 1.20)	0.25 (–0.73, 1.23)
12 mo	–0.40 (–1.29, 0.49)	0.04 (–0.84, 0.93)	0.16 (–0.79, 1.11)
24 mo	–0.72 (–1.86, 0.42)	–0.35 (–1.71, 1.01)	–0.02 (–1.18, 1.14)
Fat			
2-mo fat (g/dL)	*n =* 97	*n =* 94	*n =* 94
3 mo	–0.12 (–0.24, –0.005)^2^	–0.05 (–0.16, 0.07)	–0.20 (–0.32, –0.09)^2^
6 mo	–0.11 (–0.23, 0.004)	–0.03 (–0.13, 0.08)	–0.19 (–0.31, –0.08)^2^
12 mo	–0.09 (–0.20, 0.03)	0.02 (–0.09, 0.13)	–0.17 (–0.29, –0.06)^2^
24 mo	–0.04 (–0.18, 0.11)	0.10 (–0.05, 0.25)	–0.14 (–0.28, 0.01)
5-mo fat (g/dL)	*n =* 87	*n =* 84	*n =* 84
3 mo	—	—	—
6 mo	–0.05 (–0.19, 0.08)	–0.02 (–0.15, 0.12)	–0.13 (–0.26, 0.0001)
12 mo	–0.02 (–0.15, 0.11)	–0.02 (–0.14, 0.10)	–0.13 (–0.25, 0.001)
24 mo	0.05 (–0.11, 0.21)	–0.02 (–0.20, 0.15)	–0.12 (–0.27, 0.04)
Carbohydrate			
2-mo carbohydrate (g/dL)	*n =* 96	*n =* 93	*n =* 93
3 mo	0.26 (–0.64, 1.16)	0.41 (–0.40, 1.22)	0.28 (–0.60, 1.15)
6 mo	0.22 (–0.65, 1.09)	0.44 (–0.32, 1.20)	0.28 (–0.57, 1.13)
12 mo	0.15 (–0.73, 1.03)	0.51 (–0.27, 1.28)	0.27 (–0.58, 1.13)
24 mo	0.005 (–1.13, 1.14)	0.63 (–0.50, 1.77)	0.27 (–0.81, 1.34)
5-mo carbohydrate (g/dL)	*n =* 85	*n =* 82	*n =* 82
3 mo	—	—	—
6 mo	0.71 (–0.08, 1.49)	0.45 (–0.34, 1.24)	0.33 (–0.46, 1.11)
12 mo	0.48 (–0.27, 1.24)	–0.002 (–0.72, 0.71)	0.13 (–0.63, 0.88)
24 mo	0.04 (–0.98, 1.06)	–0.90 (–2.09, 0.29)	–0.28 (–1.26, 0.70)
Energy			
2-mo energy (kcal/dL)	*n =* 97	*n =* 94	*n =* 94
3 mo	–0.01 (–0.03, –0.001)^2^	–0.01 (–0.02, 0.01)	–0.02 (–0.04, –0.01)^2^
6 mo	–0.01 (–0.03, 0.0002)	–0.003 (–0.01, 0.01)	–0.02 (–0.03, –0.01)^2^
12 mo	–0.01 (–0.02, 0.003)	0.002 (–0.01, 0.01)	–0.02 (–0.03, –0.01)^2^
24 mo	–0.005 (–0.02, 0.01)	0.01 (–0.01, 0.03)	–0.01 (–0.03, 0.001)
5-mo energy (kcal/dL)	*n =* 87	*n =* 84	*n =* 84
3 mo	—	—	—
6 mo	–0.01 (–0.02, 0.01)	–0.002 (–0.02, 0.01)	–0.01 (–0.03, 0.001)
12 mo	–0.002 (–0.02, 0.01)	–0.002 (–0.02, 0.01)	–0.01 (–0.03, 0.001)
24 mo	0.004 (–0.01, 0.02)	–0.003 (–0.02, 0.02)	–0.01 (–0.03, 0.004)

*b* = unstandardized regression coefficient representing the change in mean outcome *z*-score per 1-unit increase in macronutrient concentration. 5-mo exposure models include timepoints 6, 12, and 24 mo only. Adjusted for birth *z*-score (weight, length, or head circumference per outcome), wealth index, maternal age, and maternal BMI.

Abbreviation: CI, confidence interval.

1 Bonferroni-corrected 97.5% CI (primary hypothesis).

2 *P* < 0.05.

Higher fat concentrations (g/dL) at 2 mo were associated with lower weight-for-age *z*-scores at 3 mo (*b:* –0.12 *z*-scores per g/dL fat; 95% CI: –0.24, –0.005) and lower head circumference-for-age *z*-scores at 3 mo (*b:* –0.20; 95% CI: –0.32, –0.09), 6 mo (*b:* –0.19; 95% CI: –0.31, –0.08), and 12 mo (*b:* –0.17; 95% CI: –0.29, –0.06). Similarly, higher energy concentrations (kcal/dL) at 2 mo were associated with lower weight-for-age *z*-scores at 3 mo (*b:* –0.01 *z*-scores per kcal/dL; 95% CI: –0.03, –0.001) and lower head circumference-for-age *z*-scores at 3 mo (*b:* –0.02; 95% CI: –0.04, –0.01), 6 mo (*b:* –0.02; 95% CI: –0.03, –0.01), and 12 mo (*b:* –0.02; 95% CI: –0.03, –0.01). There were no other significant associations between macronutrient and energy concentrations at 2 or 5 mo and infant growth *z*-scores after adjusting for confounders (Table 3).

### Macronutrient concentrations and neurodevelopment

There were no significant associations between protein concentration and cognitive (primary outcome), language, or motor domain *z*-scores at 24 mo (Table 4). Higher breast milk carbohydrate concentration at 2 mo was associated with higher motor *z*-scores (*b*: 1.08 *z*-scores per carbohydrate g/dL; 95% CI: 0.06, 2.09), whereas higher carbohydrate concentrations at 5 mo were associated with higher cognitive *z*-scores (*b*: 1.08 *z*-scores per carbohydrate g/dL; 95% CI: 0.06, 2.09). We found no other associations between breast milk macronutrient or energy concentrations at 2 or 5 mo and Bayley-III *z*-scores in either crude or adjusted analyses (Table 4, Supplemental Table 4).

**TABLE 4:** Adjusted associations between breast milk macronutrient concentration and neurodevelopmental *z*-scores at 24 mo

Exposure	Cognitive composite *z*-score	Language composite *z*-score	Motor composite *z*-score
	*b* (95% CI)	*b* (95% CI)	*b* (95% CI)
Protein (g/dL)			
2 mo *n* = 79			
	–0.30 (–1.67, 1.07)^1^	–0.03 (–1.12, 1.05)	0.23 (–0.87, 1.34)
5 mo *n* = 74			
	0.32 (–0.84, 1.48)	–0.05 (–1.10, 1.00)	0.37 (–0.70, 1.45)
Fat (g/dL)			
2 mo *n* = 80			
	–0.07 (–0.23, 0.08)	–0.02 (–0.16, 0.12)	–0.004 (–0.15, 0.14)
5 mo *n* = 74			
	0.01 (–0.17, 0.18)	0.12 (–0.03, 0.27)	–0.01 (–0.17, 0.15)
Carbohydrate (g/dL)			
2 mo *n* = 79			
	0.01 (–1.12, 1.13)	0.57 (–0.46, 1.59)	1.08 (0.06, 2.09)^2^
5 mo *n* = 72			
	1.10 (0.15, 2.05)^2^	0.32 (–0.58, 1.22)	0.77 (–0.15, 1.69)
Energy			
2 mo *n* = 80			
	–0.01 (–0.03, 0.01)	–0.002 (–0.02, 0.01)	0.001 (–0.02, 0.02)
5 mo *n* = 74			
	0.004 (–0.01, 0.02)	0.01 (–0.003, 0.03)	0.001 (–0.02, 0.02)

*b* = unstandardized regression coefficient representing the change in mean outcome *z*-score per 1-unit increase in macronutrient concentration. Adjusted for wealth index, maternal education, maternal age, and prematurity (GA proxy).

Abbreviations: CI, confidence interval; GA, gestational age.

1 Bonferroni-corrected 97.5% CI (primary hypothesis).

2 *P* < 0.05.

### Subgroup analysis: complementary feeding

For growth outcomes, there were no significant differences between infants who were exclusively breastfed at 5 mo and those that were not (Supplemental Table 5). By contrast, for neurodevelopment, the association between 5-mo carbohydrate concentration and 24-mo cognitive *z*-score—which was significant in the EBF subgroup (*b:* 1.52; 95% CI: 0.46, 2.59)—was supported by a significant interaction term in the full-sample model (*b:* 3.11; 95% CI: 0.37, 5.85; *P* = 0.027), indicating that the positive association between carbohydrate concentration and cognitive scores was significantly stronger among EBF infants compared with non-EBF infants (Supplemental Table 6).

## Discussion

In this lactation cohort, we investigated the relationship between breast milk macronutrients and child growth and neurodevelopment among a population with mother–infant dyads in rural Bangladesh. Overall breast milk concentrations of protein, fat, and energy declined from 2 to 5 mo. Length and head circumference *z*-scores also decreased over time. Contrary to our hypotheses, higher breast milk protein concentrations were not associated with improved linear growth or cognitive performance. Infants exposed to higher 2-mo fat and energy concentrations in breast milk had lower weight *z*-scores at 3 mo and head circumference *z*-scores from 3 to 12 mo, although this was not observed at 24 mo. We also found higher carbohydrate concentrations at 2 and 5 mo were associated with higher motor and cognitive performance, respectively. At 5 mo, the positive association between carbohydrates and cognition was higher among infants who were exclusively breastmilk-fed.

### Macronutrients and growth

We did not find evidence to support our hypothesis of a relationship between breast milk proteins and infant growth. Previous LMIC-based studies have also found mixed results regarding the relationship between breast milk protein concentration and growth. In a recent systematic review, authors concluded that higher protein was generally associated with improved linear growth [4]. However, the recently published mothers and infants lactation quality (MILQ) study of well-nourished mothers and infants in Bangladesh, Brazil, Denmark, and the Gambia found a negative relationship between breast milk protein concentrations and growth, albeit a positive relationship between daily protein intake and growth [34]. It is possible that the positive relationship between protein and infant growth parameters may be more related to daily intake of protein than protein concentration alone. Notably, the protein concentrations at 2 and 5 mo in our cohort approximate MILQ study reference values closely, with a mean value that decreases over time and that approximates the 50th percentile in the reference values for both time points [35]. Overall, our findings are consistent with the mixed results from other LMIC-based studies, suggesting that the concentrations alone provide an incomplete picture of protein provision.

In this cohort, infants consuming breast milk with higher concentrations of fat and energy at 2 mo had lower 3-mo weight *z*-scores and 3, 6, and 12-mo head circumference *z*-scores. Although the inverse relationship between 2-mo fat and weight/head circumference may seem surprising, other studies have demonstrated an inverse relationship between breast milk fat percentage and infant weight gain, but a concomitant positive association between breast milk volume intake and growth [36,37]. Infants consuming breast milk with higher caloric content have been found to show earlier signs of satiety and finish feeding more quickly than infants consuming lower-calorie breast milk [38]. The MILQ study also found an inverse relationship between fat and energy concentrations and milk volume, but a strong positive relationship between milk volume and infant daily macronutrient intake [39]. Overall, it seems biologically implausible that the inverse relationship between fat/energy concentrations and weight/head circumference is causal in our cohort.

However, this negative association begs further investigation into the relationships between macronutrient concentrations, breast milk intake volumes, and infant growth in future studies. Breast milk concentrations alone represent an incomplete picture of contributors to infant growth, whereas accounting for milk intake volumes, combined with composition, provides a more comprehensive picture of the relationship between breast milk and infant growth. Our study occurred among a population with high rates of exclusive and extended breastfeeding, but early complementary feeding and mixed feeding with formula—common practices in many global locations—also play an important role in nutrition-driven infant outcomes.

Notably, many factors affect breast milk fat concentrations. We used a mid-feed sample with a standardized operating procedure of a consistent, measured time for an infant to directly breastfeed before hand expression. The goal of our standardized procedure was to maximize comparability between women within our cohort to the best extent possible within the cultural limitations of our setting, where a full expression would not be acceptable. In a systematic review of breast milk collection methods, authors cite 24-h full-feed collection as the reference standard for breast milk collection where feasible, although acknowledging that this “reference standard” was primarily used in older studies and may be excessively burdensome to participants [34]. This burden may restrict the study populations who are willing to participate and limit the generalizability of findings [40]. Overall, standardization of methods with regard to time of day, time since the last feed, collection mode (i.e., hand expression), and sampling procedures are critical to enable reproducible results regarding fat concentrations and breast milk.

### Macronutrients and neurodevelopment

Contrary to our hypotheses, we did not find a relationship between protein concentrations and neurodevelopment. Virtually all previous studies of this relationship have focused on preterm infants with higher intrinsic protein requirements [41,42]. The relationship between proteins and amino acid intake has been identified as a gap in the field of breast milk and neurodevelopment [43]. Although our findings suggest that the protein concentrations may not be associated with neurodevelopment at 24 mo, further analyses are needed to better characterize the relationship between daily breast milk protein intake and child development.

Higher 2- and 5-mo carbohydrate concentrations were associated with higher motor and cognitive composite *z*-scores, respectively. Total carbohydrate concentrations in breast milk represent a combination of lactose (∼80%) and human milk oligosaccharides (HMOs) (∼20%). Lactose is critical for glucose regulation. It is possible that higher concentrations of lactose may result in lower rates of hypoglycemia, a relatively common condition in growth-restricted infants, such as many of the infants enrolled in our study, which is known to negatively impact neurodevelopment [44]. However, lactose is one of the most stable elements in breast milk with very limited variability between individuals, so this mechanism is likely a minor contributor to the associations we see with neurodevelopment [45]. HMOs comprise the remainder of measured carbohydrate and are nondigestible, prebiotic substrates with concentrations that vary widely between individuals [46]. Higher concentrations of HMOs in the first months of breastfeeding have been associated with improved neurodevelopmental outcomes at 18–24 mo [47]. Given the narrow SDs of carbohydrates observed in our cohort, it may be that variability in HMO concentration is driving the positive association between carbohydrates and Bayley-III cognitive and motor *z*-scores. Interestingly, the positive association between carbohydrate concentrations and infant cognitive performance was stronger in the subgroup of infants who were exclusively breastfed at 5 mo as compared with those who received early complementary feeding, strengthening this result. Limited data are available regarding the relationship between breast milk total carbohydrate concentrations and neurodevelopment, but our findings suggest that this may be a fruitful area for further investigation.

### Limitations and strengths

Our study has certain limitations. Although a full expression would have been optimal for the measurement of fat and calculation of energy content, it was not culturally acceptable at the time of the study, and so mid-feed milk was collected instead. We were also limited to a single expression of milk at each time point because of the lack of feasibility for 24-h collection among women living in this rural area with no access to refrigeration. This is common in LMIC-based milk composition studies [48]. Lastly, our sample size was modest, decreasing statistical power and widening the CIs around our estimates.

Our findings should be interpreted as hypothesis-generating. They may be used to inform the design of future studies and analyses and may not be generalizable to other populations. For example, the population in rural Sylhet, Bangladesh has a unique infant feeding profile with high rates of EBF and low rates of formula use, limiting the ability to extend conclusions to populations with high rates of mixed feeding with both breastmilk and formula. Rates of maternal undernutrition/low BMI and infant growth restriction are extremely high and may result in child growth patterns that are not generalizable to a well-nourished population. Larger longitudinal studies of human milk composition among nutritionally vulnerable populations with high rates of maternal undernutrition and fetal growth restriction are needed to confirm our findings regarding variation in breast milk macronutrient concentrations and child growth and developmental outcomes.

Our study had many strengths. This study contributes to an extremely limited evidence base on breast milk macronutrient composition and neurodevelopment among term infants. To our knowledge, this is one of the first studies to examine this relationship. Our data are enriched by studying all macronutrients and energy within a single cohort at 2 time points in lactation, allowing us to explore the biology of breast milk over time. Our study is also strengthened by accurate ultrasound-based GA dating, which is unusual in rural Bangladesh, where access to accurate GA determination is limited. Accurate GA is crucial for assigning growth *z*-scores based on postmenstrual age. Finally, the Bayley-III is a well-established standard of measuring neurodevelopment, which was rigorously administered by highly skilled study psychologists who had undergone training using a culturally relevant, validated version of the Bayley-III. This study is unique in our ability to estimate the relationship between breast milk macronutrient concentrations and infant neurodevelopment using the Bayley, which is often considered a reference standard metric of infant neurodevelopment.

In conclusion, in this prospective cohort of mothers and infants in rural Bangladesh, protein concentrations were not associated with length or cognition during infancy and early childhood. Fat and energy concentrations were inversely associated with head circumference growth throughout the first year of life. To our knowledge, this is among the first studies to examine breast milk macronutrient concentrations in relation to child neurodevelopment, and we found a positive association with carbohydrate concentration. Future research may focus on confirming or replicating these findings and examining additional factors, such as milk intake volume, that may further our understanding of growth and neurodevelopment patterns observed among breastfed infants.

## Author contributions

The authors’ responsibilities were as follows – KN, SD, RS, SC, ACCL: designed research (project conception, development of overall research plan, and study oversight); ACCL, CA, SA, SR, AB, RK, NHC, FT, TH: conducted research (hands-on conduct of the experiments and data collection); MBB, SA, SR, AB, RK: provided essential reagents, or provided essential materials (applies to authors who contributed by providing animals, constructs, databases, etc., necessary for the research); KN, SD, SC, ALE, URP, CMD: analyzed data or performed statistical analysis; KN: wrote the paper; KN, RS, ACCL: took primary responsibility for final content; and all authors: read and approved the final manuscript.

## Data availability

Data described in the manuscript, code book, and analytic code will be made available on request pending approval by the Projahnmo study team.

## Declaration of generative AI and AI-assisted technologies in the writing process

The author(s) declare that no generative AI or AI-assisted technologies were used in the writing of this manuscript.

## Funding

This research was funded in part by: The Bill and Melinda Gates Foundation (OPP1211778), National Institute of Child Health and Development (K23 HD091390 and K23 HD118179), Thrasher Research Fund (01837), World Health Organization (I64438), and Brigham Research Institute, University of North Carolina at Chapel Hill School of Medicine. The sponsors played no role in the publication.

## Conflict of interest

The authors report no conflicts of interest.
