# Supplementary material for: Breast Milk Macronutrients and Child Growth and Neurodevelopment in a Lactation Cohort in Rural Bangladesh
Source: Curr Dev Nutr. 2026 Sep 16;10(10):109531. doi: 10.1016/j.cdnut.2026.109531 (PMC13676091; doi:10.1016/j.cdnut.2026.109531)
Supplement: Supplementary file 1 [file mmc1.docx]

**Breast milk macronutrients and child growth and neurodevelopment in a lactation cohort in rural Bangladesh**

**First author: Krysten North**

Supplementary Material

**Contents**

[**Supplementary Table 1. Demographics by retention group (median + IQR and Wilcoxon rank sum p-value)**	2](#_Toc232426907)

[**Supplementary Table 2.** **Population characteristics of 99 mother-infant dyads (mean (SD) or Median [IQR])**	3](#_Toc232426908)

[**Supplementary Table 3. Unadjusted associations between breast milk macronutrient concentration and infant growth z-scores**	4](#_Toc232426909)

[**Supplementary Table 4. Unadjusted associations between breast milk macronutrient concentration and Bayley-III neurodevelopmental z-scores at 24 months**	6](#_Toc232426910)

[**Supplementary Table 5. Adjusted associations — exclusively breastfed subgroup — growth z-scores**	7](#_Toc232426911)

[**Supplementary Table 6. Adjusted associations — exclusively breastfed subgroup — neurodevelopmental z-scores at 24 months**	8](#_Toc232426912)

| **Supplementary Table 1. Demographics by retention group (median + IQR and Wilcoxon rank sum p-value)** | | | |
| --- | --- | --- | --- |
| Variable | 2 month milk only | 2 + 5 month milk | p-value |
| **Maternal characteristics** | | | |
| Maternal age (years)* | 25.4 ± 5.9 | 23.6 ± 4.1 | 0.18 |
| Maternal education (# years)* | 6.5 ± 3.0 | 7.1 ± 2.9 | 0.50 |
| Parity | 2.0 (1.0–3.0) | 2.0 (1.0–3.0) | 0.65 |
| Maternal BMI at enrollment (kg/m^2^) | 19.8 (18.8–21.5) | 19.2 (17.9–21.6) | 0.46 |
| Age of introduction at complimentary feeds (months) | 5.5 (5.0–6.0) | 5.0 (4.0–6.0) | 0.55 |
| **Infant characteristics** | | | |
| Male sex** | 6/13 (46.2%) | 42/83 (50.6%) | 1.00 |
| GA at delivery (weeks) | 39.3 (39.0–39.7) | 39.6 (38.6–40.6) | 0.57 |
| Birthweight (kg)* | 2.8 ± 0.2 | 2.7 ± 0.4 | 0.44 |
| Abbreviations: BMI body mass index; GA gestational age *mean (SD) and t test reported **n (%) and Fisher’s exact p-value reported | | | |

| **Supplementary Table 2.** **Population characteristics of 99 mother-infant dyads (mean (SD) or Median [IQR])** | | |
| --- | --- | --- |
|  | **Lactation cohort** | **Parent pregnancy cohort^1,2^** |
| **Maternal Characteristics** | **n= 99** | n= 2982 |
| Age (years) | 24.0 [20.0-27.0] | 24 [20-27] |
| Education (# years) | 6.9 (2.9) | 6.5 (3.0) |
| Maternal MUAC at enrollment (cm) | 23.1 (2.3) | 22.9 (2.4) |
| **Infant Characteristics** |  |  |
| GA at delivery (weeks) | 39.6 [38.9-40.4] | 40.0 (0.1) |
| Preterm delivery (<37 weeks) (n, %) | 6.0 (6.1%) | 9.20% |
| Birthweight (g) | 2747 (358) | 2721 (469) |

Abbreviations: BMI = body mass index; GA gestational age

| **Supplementary Table 3. Unadjusted associations between breast milk macronutrient concentration and infant growth z-scores** | | | |
| --- | --- | --- | --- |
|  | Weight-for-age z-score | Length-for-age z-score | Head circumference-for-age z-score |
| Age | *b* (95% CI) | *b* (95% CI) | *b* (95% CI) |
| Protein | | | |
| 2-month protein (g/dL) n: WTZ=96, LAZ=97, HCZ=97 | | | |
| 3 months | 0.005 (-1.00, 1.01) | 0.18 (-0.83, 1.20) | 0.90 (-0.09, 1.88) |
| 6 months | -0.02 (-0.99, 0.95) | 0.10 (-0.88, 1.07) | 0.94 (-0.02, 1.90) |
| 12 months | -0.07 (-1.04, 0.90) | -0.08 (-1.19, 1.03)* | **1.02 (0.07, 1.98)** |
| 24 months | -0.17 (-1.37, 1.03) | -0.43 (-1.67, 0.81) | **1.19 (0.06, 2.33)** |
| 5-month protein (g/dL) n: WTZ=87, LAZ=87, HCZ=87 | | | |
| 3 months | — | — | — |
| 6 months | -0.21 (-1.17, 0.75) | -0.17 (-1.22, 0.88) | 0.26 (-0.72, 1.25) |
| 12 months | -0.38 (-1.31, 0.56) | -0.35 (-1.34, 0.64) | 0.27 (-0.70, 1.23) |
| 24 months | -0.71 (-1.88, 0.47) | -0.71 (-2.12, 0.70) | 0.27 (-0.89, 1.43) |
| Fat | | | |
| 2-month fat (g/dL) n: WTZ=97, LAZ=98, HCZ=98 | | | |
| 3 months | -0.11 (-0.23, 0.02) | -0.05 (-0.18, 0.07) | -0.10 (-0.23, 0.02) |
| 6 months | -0.10 (-0.22, 0.02) | -0.03 (-0.15, 0.09) | -0.10 (-0.22, 0.02) |
| 12 months | -0.07 (-0.19, 0.05) | 0.02 (-0.11, 0.14) | -0.09 (-0.21, 0.03) |
| 24 months | -0.02 (-0.17, 0.13) | 0.11 (-0.05, 0.27) | -0.07 (-0.22, 0.08) |
| 5-month fat (g/dL) n: WTZ=87, LAZ=87, HCZ=87 | | | |
| 3 months | — | — | — |
| 6 months | -0.04 (-0.18, 0.10) | 0.04 (-0.11, 0.19) | -0.07 (-0.22, 0.07) |
| 12 months | -0.01 (-0.14, 0.13) | 0.04 (-0.11, 0.18) | -0.07 (-0.21, 0.07) |
| 24 months | 0.06 (-0.11, 0.22) | 0.03 (-0.16, 0.22) | -0.05 (-0.22, 0.11) |
| Carbohydrate | | | |
| 2-month carbohydrate (g/dL) n: WTZ=96, LAZ=97, HCZ=97 | | | |
| 3 months | 0.19 (-0.73, 1.12) | -0.12 (-1.06, 0.81) | -0.14 (-1.09, 0.81) |
| 6 months | 0.16 (-0.74, 1.05) | -0.09 (-0.98, 0.81) | -0.15 (-1.08, 0.78) |
| 12 months | 0.09 (-0.82, 0.99) | -0.01 (-0.92, 0.89) | -0.17 (-1.10, 0.76) |
| 24 months | -0.06 (-1.22, 1.10) | 0.14 (-1.09, 1.36) | -0.22 (-1.35, 0.92) |
| 5-month carbohydrate (g/dL) n: WTZ=85, LAZ=85, HCZ=85 | | | |
| 3 months | — | — | — |
| 6 months | 0.62 (-0.21, 1.44) | 0.54 (-0.35, 1.42) | 0.19 (-0.66, 1.04) |
| 12 months | 0.39 (-0.40, 1.18) | 0.11 (-0.72, 0.93) | -0.01 (-0.83, 0.81) |
| 24 months | -0.06 (-1.10, 0.98) | -0.75 (-2.00, 0.50) | -0.42 (-1.45, 0.60) |
| Energy | | | |
| 2-month energy (kcal/dL) n: WTZ=97, LAZ=98, HCZ=98 | | | |
| 3 months | -0.01 (-0.03, 0.002) | -0.01 (-0.02, 0.01) | -0.01 (-0.03, 0.003) |
| 6 months | -0.01 (-0.02, 0.002) | -0.004 (-0.02, 0.01) | -0.01 (-0.02, 0.003) |
| 12 months | -0.01 (-0.02, 0.01) | 0.001 (-0.01, 0.01) | -0.01 (-0.02, 0.004) |
| 24 months | -0.003 (-0.02, 0.01) | 0.01 (-0.01, 0.03) | -0.01 (-0.02, 0.01) |
| 5-month energy (kcal/dL) n: WTZ=87, LAZ=87, HCZ=87 | | | |
| 3 months | — | — | — |
| 6 months | -0.004 (-0.02, 0.01) | 0.004 (-0.01, 0.02) | -0.01 (-0.02, 0.01) |
| 12 months | -0.001 (-0.02, 0.01) | 0.003 (-0.01, 0.02) | -0.01 (-0.02, 0.01) |
| 24 months | 0.005 (-0.01, 0.02) | 0.002 (-0.02, 0.02) | -0.01 (-0.02, 0.01) |
| *b* = unstandardized regression coefficient representing the change in mean outcome z-score per 1-unit increase in macronutrient concentration. Bold = p < 0.05. 5-month exposure models include timepoints 6, 12, and 24 months only. Unadjusted models. * Bonferroni-corrected 97.5% CI (primary hypothesis). | | | |

| **Supplementary Table 4. Unadjusted associations between breast milk macronutrient concentration and Bayley-III neurodevelopmental z-scores at 24 months** | | | |
| --- | --- | --- | --- |
|  | Cognitive composite z-score | Language composite z-score | Motor composite z-score |
| Exposure | *b* (95% CI) | *b* (95% CI) | *b* (95% CI) |
| Protein | | | |
| 2-month protein (g/dL) n = 79 | | | |
|  | -0.38 (-1.74, 0.98)* | 0.05 (-1.03, 1.13) | 0.21 (-0.88, 1.29) |
| 5-month protein (g/dL) n = 74 | | | |
|  | 0.48 (-0.67, 1.63) | 0.05 (-1.01, 1.11) | 0.41 (-0.65, 1.47) |
| Fat | | | |
| 2-month fat (g/dL) n = 80 | | | |
|  | -0.07 (-0.22, 0.08) | -0.003 (-0.14, 0.14) | 0.004 (-0.14, 0.14) |
| 5-month fat (g/dL) n = 74 | | | |
|  | -0.01 (-0.18, 0.15) | 0.11 (-0.04, 0.26) | -0.01 (-0.16, 0.14) |
| Carbohydrate | | | |
| 2-month carbohydrate (g/dL) n = 79 | | | |
|  | -0.16 (-1.28, 0.97) | 0.43 (-0.59, 1.45) | 0.94 (-0.06, 1.94) |
| 5-month carbohydrate (g/dL) n = 72 | | | |
|  | 0.82 (-0.12, 1.76) | 0.10 (-0.80, 0.99) | 0.56 (-0.34, 1.46) |
| Energy | | | |
| 2-month energy (kcal/dL) n = 80 | | | |
|  | -0.01 (-0.02, 0.01) | -0.000003 (-0.02, 0.02) | 0.002 (-0.01, 0.02) |
| 5-month energy (kcal/dL) n = 74 | | | |
|  | 0.002 (-0.02, 0.02) | 0.01 (-0.003, 0.03) | 0.001 (-0.02, 0.02) |
| *b* = unstandardized regression coefficient representing the change in mean outcome z-score per 1-unit increase in macronutrient concentration. Bold = p < 0.05. n = participants included in model, shown in row heading. Unadjusted models. * Bonferroni-corrected 97.5% CI (primary hypothesis). | | | |

| **Supplementary Table 5. Adjusted associations — exclusively breastfed subgroup — growth z-scores** | | | |
| --- | --- | --- | --- |
|  | Weight-for-age z-score | Length-for-age z-score | Head circumference-for-age z-score |
| Age | *b* (95% CI) | *b* (95% CI) | *b* (95% CI) |
| Protein | | | |
| 5-month protein (g/dL) n: WTZ=56, LAZ=55, HCZ=55 | | | |
| 6 months | -0.53 (-1.86, 0.80) | -0.004 (-1.28, 1.27) | -0.35 (-1.52, 0.82) |
| 12 months | -0.73 (-2.03, 0.56) | -0.86 (-2.07, 0.35) | -0.35 (-1.49, 0.78) |
| 24 months | -1.14 (-2.79, 0.52) | **-2.57 (-4.49, -0.66)** | -0.36 (-1.85, 1.12) |
| Fat | | | |
| 5-month fat (g/dL) n: WTZ=56, LAZ=55, HCZ=55 | | | |
| 6 months | -0.03 (-0.21, 0.14) | -0.004 (-0.17, 0.16) | -0.05 (-0.20, 0.10) |
| 12 months | -0.02 (-0.19, 0.15) | -0.03 (-0.19, 0.12) | -0.06 (-0.21, 0.08) |
| 24 months | -0.01 (-0.20, 0.19) | -0.09 (-0.30, 0.12) | -0.08 (-0.25, 0.09) |
| Carbohydrate | | | |
| 5-month carbohydrate (g/dL) n: WTZ=55, LAZ=54, HCZ=54 | | | |
| 6 months | **1.08 (0.25, 1.90)** | 0.52 (-0.34, 1.38) | 0.48 (-0.29, 1.25) |
| 12 months | **0.92 (0.13, 1.71)** | 0.13 (-0.67, 0.92) | 0.33 (-0.42, 1.07) |
| 24 months | 0.61 (-0.45, 1.68) | -0.66 (-1.93, 0.61) | 0.02 (-0.96, 1.00) |
| Energy | | | |
| 5-month energy (kcal/dL) n: WTZ=56, LAZ=55, HCZ=55 | | | |
| 6 months | -0.003 (-0.02, 0.02) | -0.001 (-0.02, 0.02) | -0.01 (-0.02, 0.01) |
| 12 months | -0.003 (-0.02, 0.02) | -0.01 (-0.02, 0.01) | -0.01 (-0.02, 0.01) |
| 24 months | -0.001 (-0.02, 0.02) | -0.01 (-0.04, 0.01) | -0.01 (-0.03, 0.01) |
| *b* = unstandardized regression coefficient representing the change in mean outcome z-score per 1-unit increase in macronutrient concentration. Bold = p < 0.05. 5-month exposure models include timepoints 6, 12, and 24 months only. Adjusted for birth z-score (weight, length, or head circumference per outcome), wealth index, maternal age, and maternal BMI. Restricted to infants exclusively breastfed (had not received formula, animal milk, or rice) at the 5-month visit. | | | |

| **Supplementary Table 6. Adjusted associations — exclusively breastfed subgroup — neurodevelopmental z-scores at 24 months** | | | |
| --- | --- | --- | --- |
|  | Cognitive composite z-score | Language composite z-score | Motor composite z-score |
| Exposure | *b* (95% CI) | *b* (95% CI) | *b* (95% CI) |
| Protein | | | |
| 5-month protein (g/dL) n = 46 | | | |
|  | 0.26 (-1.62, 2.15) | -0.76 (-2.44, 0.91) | 0.21 (-1.57, 1.99) |
| Fat | | | |
| 5-month fat (g/dL) n = 46 | | | |
|  | -0.003 (-0.23, 0.22) | 0.04 (-0.17, 0.24) | -0.05 (-0.27, 0.16) |
| Carbohydrate | | | |
| 5-month carbohydrate (g/dL) n = 45 | | | |
|  | **1.52 (0.46, 2.59)** | 0.36 (-0.68, 1.40) | 0.63 (-0.44, 1.71) |
| Energy | | | |
| 5-month energy (kcal/dL) n = 46 | | | |
|  | 0.003 (-0.02, 0.03) | 0.004 (-0.02, 0.03) | -0.003 (-0.03, 0.02) |
| *b* = unstandardized regression coefficient representing the change in mean outcome z-score per 1-unit increase in macronutrient concentration. Bold = p < 0.05. n = participants included in model, shown in row heading. Adjusted for wealth index, maternal education, maternal age, and prematurity (GA proxy). Restricted to infants exclusively breastfed (had not received formula, animal milk, or rice) at the 5-month visit. | | | |

Works Cited in Supplementary Material:

Aftab F, Ahmed I, Ahmed S, Ali SM, Amenga-Etego S, Ariff S, et al. Direct maternal morbidity and the risk of pregnancy-related deaths, stillbirths, and neonatal deaths in South Asia and sub-Saharan Africa: A population-based prospective cohort study in 8 countries. PLoS Med. 2021 Jun 28;18(6):e1003644.

Lee ACC, Whelan R, Bably NN, Schaeffer LE, Rahman S, Ahmed S, Moin SMI, Begum N, Quaiyum MA, Rosner B, Litch JA, Baqui AH, Wylie BJ. Prediction of gestational age with symphysis-fundal height and estimated uterine volume in a pregnancy cohort in Sylhet, Bangladesh. BMJ Open. BMJ; 2020 Mar. 1;10(3):e034942.
